# Targeting Cancer with Phytochemicals via Their Fine Tuning of the Cell Survival Signaling Pathways

**DOI:** 10.3390/ijms19113568

**Published:** 2018-11-12

**Authors:** Salvatore Chirumbolo, Geir Bjørklund, Roman Lysiuk, Antonio Vella, Larysa Lenchyk, Taras Upyr

**Affiliations:** 1 Department of Neuroscience, Biomedicine and Movement Sciences, University of Verona, 37134 Verona, Italy; 2 Scientific Secretary-Council for Nutritional and Environmental Medicine (CONEM), 8610 Mo i Rana, Norway; 3 Council for Nutritional and Environmental Medicine (CONEM), 8610 Mo i Rana, Norway; bjorklund@conem.org; 4 Department of Pharmacognosy and Botany, DanyloHalytskyLviv National Medical University, 79007 Lviv, Ukraine; pharmacognosy.org.ua@ukr.net; 5 AOUI Verona, University Hospital, Section of Immunology, 37134 Verona, Italy; antonio.vella@univr.it; 6 Department of Chemistry of Natural Compounds, National University of Pharmacy, 61168 Kharkiv, Ukraine; larysa.lenchyk@gmail.com; 7 Department of Pharmacognosy, National University of Pharmacy, 61168 Kharkiv, Ukraine; upyrtaras@gmail.com

**Keywords:** phytochemicals, flavonoids, cancer, mitochondria, apoptosis

## Abstract

The role of phytochemicals as potential prodrugs or therapeutic substances against tumors has come in the spotlight in the very recent years, thanks to the huge mass of encouraging and promising results of the in vitro activity of many phenolic compounds from plant raw extracts against many cancer cell lines. Little but important evidence can be retrieved from the clinical and nutritional scientific literature, where flavonoids are investigated as major pro-apoptotic and anti-metastatic compounds. However, the actual role of these compounds in cancer is still far to be fully elucidated. Many of these phytochemicals act in a pleiotropic and poorly specific manner, but, more importantly, they are able to tune the reactive oxygen species (ROS) signaling to activate a survival or a pro-autophagic and pro-apoptosis mechanism, depending on the oxidative stress-responsive endowment of the targeted cell. This review will try to focus on this issue.

## 1. Introduction

The ability of phytochemicals to prevent cancer has been long claimed as the most outstanding potential of these plant-derived molecules, despite the fact that quite the whole bulk of these substances is made of toxic compounds [1,2,3,4]. However, the purported anticancer activity of many phenolics produced in the natural world, accounting for more than 5000 different chemically characterized compounds in edible plants [5], has been reported to fundamentally depend on their antioxidant property [6]. Notwithstanding, the ability of phytochemicals to induce the expression of the Nrf2-ARE signaling pathway [7,8,9], despite the evidence that all stressors can activate the Nrf2-ARE system [10], the potential of these plant-derived phenolic substances to promote, enhance and trigger the expression and the activity of superoxide dismutase (SOD), catalase (CAT) and glutathione peroxydase (GPX) [11], represent a fundamental hallmark to trust these substances as possible chemopreventive molecules [12,13,14,15,16]. How phytochemicals can counteract tumors is still far to be fully elucidated. Phytochemicals have a huge panoply of intracellular targets and their interaction with many of them is yet puzzling to date and particularly intriguing [17]. A first issue to be focused on is the relationship between mitochondria, reactive oxygen species (ROS) and cancer [18,19,20,21]. Plant-derived phytoestrogens, such as catechins, can induce mitochondrial biogenesis, thus restoring mitochondria function, via the induction of survival signaling systems activated during mitohormesis, such as AMPK/AAK-2, SIRT1/SIR-2.1 and FOXO/DAF-16, in *C. elegans* [22]. Targeting mitochondria should demonstrate also the ability of these molecules to tune cell morphogenesis and improve mitochondria function and biogenesis [23,24,25]. Actually, alterations in the mitochondrial dynamics modulate some type of tumors. For example, the dysregulated mitochondrial fusion by Mfn2 knockdowns suppresses the rate of oxygen consumption in melanoma cells, suggesting that mitochondrial dynamics, i.e., the rate of fission and fusion, modulate cell migration and progression in this type of cancer [26]. Dihydromyricetin is able to reverse mitochondrial dysfunction, which should be mediated by PGC-1α/TFAM and PGC-1α/mfn2 signaling pathways, therefore ameliorating mitochondria dynamics [27]. Mitochondria dysfunction is a typical hallmark of many cancers and the ability of phytochemicals to restore it appears quite fundamental [28,29,30]. The fìne regulation of the survival process in a cell involves a series of signaling pathways that not only encompasses the enzymatic endowment for ROS scavenging but also the complex machinery modulation of the crosstalk between mitochondria and other organelles leading to the autophagy/apoptosis balance [31,32,33].

The role of phytochemicals in this context is particularly interesting [34,35].

Phytochemicals not only may counteract cancer malignancy and progression but can induce tumor cells necroptosis, besides apoptosis [36,37]. Furthermore, the role of autophagy in cancer development has been extensively reviewed in recent years [38]. Although autophagy would lead to a suppression of tumorigenesis, some circumstances showed an opposite action on cancer [38,39]. Therefore, the ability of phytochemicals to target cellular autophagy as an approach in using the natural substances as chemopreventive compounds should be considered with particular attention, despite the many encouraging results [40,41,42]. Their activity might also target intracellular calcium signaling and endoplasmic reticulum (ER) stress [43,44], which exerts a major role in the mitochondria-mediated tuning of the many cell survival functions [45]. A role in maintaining the mitochondria–ER stress homeostasis has been recently attributed to Lon proteases (LONPs), where LONP is a protein complex made by a homo-hexameric ring-shaped structure with a serine–lysine catalytic dyad, which is highly conserved in both prokaryotic and eukaryotic organisms [46,47]. LONPs are upregulated during ER stress, via the activation of the PERK-ATF4 signaling pathway [48,49], which may be targeted by flavonoids [50,51,52]. In this perspective, plant-derived polyphenols might target many anti-oxidant cell signaling systems, which exert a major role in mitochondria biogenesis and mitochondria–ER stress homeostasis. The close interaction between mitochondria and ER may be regulated by caveolin-1, which is located at the mitochondria/ER interface where it impairs the remodeling of the mitochondria–ER relationship by making mitochondria non responsive to ER stress via the dampening of the calcium signaling [53,54]. This mechanism is counterbalanced by the PKA-DRP1-mediated signaling [54,55], which is targeted by flavonoids [56]. In cancer cells, this homeostasis can be profoundly perturbed and the activity of flavonoids can be functionally inverted with respect to the one acting on normal, non-cancerous cells [57]. Actually, tumors have a different stress response with respect to non tumoral cells, so that any therapic approach must take into account this issue [58,59].

In this review, we will attempt to elucidate the very recent novelties in the field of cancer prevention and therapy using nature-derived phytochemicals.

## 2. Insights on the Role of Flavonoids in Cancer

### 2.1. Flavonoids and Apoptosis

Table 1 summarizes some of the very recent results about the flavonoids ability in inhibiting cancer development and malignancy [60,61,62,63,64,65,66,67,68,69,70,71,72,73,74,75,76,77,78,79,80,81,82,83,84,85,86,87]. Many of these molecules act against cancer cells by promoting and activating apoptosis. The signaling pathways through which flavonoids induce apoptosis in cancerous cells are various. Besides the effect on Bax, Bcl-2 and caspases, a further possibility is represented by the inhibition of fatty acid synthase (FAS) exerted by a great number of flavonoids, such as epigallocatechin-3-gallate (EGCG), luteolin, quercetin, kaempferol, apigenin, and taxifolin, which exert their anti-lipogenic activities against many human tumors [88,89]. FAS is over-expressed in many human epithelial cancers and also in breast tumors. Its inhibition, causing the accumulation of malonyl-CoA, leads to the upregulation of ceramide levels and the inhibition of carnitine palmitoyltransferase-1, therefore inducing the expression of the pro-apoptotic genes BNP3, TRAIL and DAPK2 and causing apoptosis [90]. Interestingly, FAS inhibition causes a massive ROS upregulation, which has been reported as a key factor in promoting cancer cell apoptosis, curiously suggesting that antioxidants promote oxidative stress to kill cancerous cells [91]. Actually, flavonoids inhibit apoptosis in non cancerous cells, probably because of their induction of eustress and hence of the survival response from cells [17,92], a mechanism closely depending on the flavonoids dose [93] and their interaction with ROS as signaling molecules [17,94]. Cancer cells are characterized by a high degree of oxidative stress and ROS production, and they possess a high metabolic and peroxysomal activity, often leading to mitochondrial dysfunction and an enhanced activity of lypooxygenases, thymidine phosphorylases, oxydases and cyclooxygenases [95]. However, despite the expected need in ROS scavenging enzymes, flavonoids exert their anticancer activity very rarely with the sole increase in the anti-oxidant machinery. More complex coupled mechanisms would occur. For example, esculetin and quercetin act finely tuning the redox homeostasis in NB4 leukemia cells [96]. Paradoxically, 25 μM quercetin increased NF-κB p65 in the nucleus and reduced it in the cytosol, whereas it reduced Nrf2 in the nucleus and enhanced it in the cytosol [96]. Furthermore, esculetin, in contrast with quercetin, increases the level of the superoxide dismutase (SOD) expression [96]. A fine tuning in the cell, where flavonoids may exert an apparently contradictory action, does exist. Moreover, the hypothesis that cancer might be closely associated with an imbalance in the energy homeostass, therefore including also ROS signaling and mitochondria oscillation, has been recently addressed [17,97].

Most of flavonoids trigger the apoptotic pathways in cancer cells via their fundamental signaling pathways. The isoflavone analog phenoxodiol induces apoptosis in renal cancer by the inhibition of the Akt pathway, whereas apoptosis is induced in non cancerous cells by phosphorylation of Akt [98,99]. The induction of apoptosis in cancer cells via the PI3K/Akt signaling pathway is held by a wide spectrum of phytochemicals. Luteolin induces a caspase-dependent apoptosis in human hepatocellular carcinoma by inhibiting the Akt phosphorylation [100], and the flavonol glucoside icanin (40 μM) induces apoptosis via a ROS-mediated damage on mitochondria membrane potential by suppressing the PI3K/Akt and STAT3 signaling pathways [101]. Furthermore, baicalein, by acting on the PI3K/Akt/NF-κB, enhances the sensitivity to cisplatin of A549 lung adenocarcinoma cell line [102].Flavonoids, acting on cells via the PI3K/Akt pathway, should regulate cell survival and apoptosis at a post-mitochondrial level, i.e., downstream of the mitochondria cytochrome c release and before the activation of caspase 9 [103]. ROS have a fundamental role in the Akt-mediated signaling leading to apoptosis, as they activate the Akt/ASK1/p38MAPK pathway, causing a modulation of ASK1 dephosphorylation, which subsequently activates p38MAPK and downregulating p21 (Cip1), leading to apoptosis [104,105]. The ability of flavonoids to cause apoptosis in cancer cells via the Akt pathway may be paradoxically mediated by a rapid imbalance in the intracellular ROS homeostass or, more probably, by the complex relationship between ASK1 and NF-κB, where IKK has a major control on the ASK1-JNK axis, associating IKK with ROS and ER stress [106]. Actually, despite the previous belief about the general increase in NF-κB expression in cancer, recent reports showed that tumors differentially express specific subunits of the NF-κB pathway, suggesting the possible existence of a finely regulated tuning towards apoptosis by different flavonoids in different target cells [107]. Actually, 3,4’,7-O-trimethyl quercetin, a derivative of quercetin, induces apoptosis in ovarian cancer cell lines CRL-1978, CRL-11731, SK-OV-3, following three different target pathways, depending on the cell type [108].

The widespread knowledge about flavonoids in cancer is that these compounds are generally very able to have anti-oxidant and anti-inflammatory activities, to induce ROS-scavenging enzymes and CYP-mediated detoxyfication, to induce cell cycle arrest, apoptosis and autophagy, and to inhibit proliferation, migration and malignancy [109]. Apoptosis is generally activated by disturbing the mitochondria–ER stress balance and ROS homeostasis, accessing this machinery via the inhibition of many survival pathways. Actually, pathways by which flavonoids induce apoptosis are altogether related to cell survival processes. Glabridin causes apoptosis in oral cancer cells via the JNK1/2 pathway, which with NF-κB is involved in cell survival, apoptosis, inflammation and angiogenesis [110,111]; hesperedin induces apoptosis in endometrial carcinoma by downregulating the estrogen receptor relationship via the ERK/MAPK pathway [112]; apigenin triggers apoptosis and autophagy in hepatocellular carcinoma cells via the PI3K/Akt/mTOR signaling pathway [113]; chrysin and other fundamental components of propolis, such as the phenolic acids caffeic, ferulic and α-coumaric acid, induce a proline dehydrogenase/proline oxidase-dependent apoptosis in human tnongue squamous cell carcinoma [114]. The panoply of different targets by which flavonoids induce apoptosis may be a consequence of their modulatory role in balancing the apoptosis/necrosis ratio [115].

Research on apoptosis and autophagy caused by phytochemicals seems to be mostly polarized on certain types of cancer, i.e., breast, prostate and colon cancer and more frequently involving curcumin, flavonoids, and resveratrol [116]. Curcumin and resveratrol also induce apoptosis via the same survival pathways targeted by several flavonoids, e.g., PI3K/Akt [117,118], MAPK/JAK2/STAT3 [119,120], p38MAPK/ERK1/2/JNK [121,122], Wnt/β-catenin [123,124], NF-κB [125,126]. Besides these major pathways, phytochemicals are able to induce apoptosis in cancer cells, targeting many further signaling and biochemical mechanisms. For example, resveratrol is able to target cellular FLICE-inhibitory protein (c-FLIP), which is a master tuner in the inhibition of apoptosis with the death receptors TNF-R1, Dr5, DR4 and Fas. In lung cancer cells, resveratrol causes p-Akt and c-FLIP downregulation, inducing apoptosis along with an increase in the production of ROS and hydrogen peroxide-elicited Bid, activation of PARP and caspase 8, and downregulation of pEGFR and NF-κB protein expression [127,128].

The most common route through which flavonoids induce apoptosis in cancer cells, i.e., the PI3K/Akt pathway, is probably the major signaling mechanism leading to cell survival gene expression with different approaches, e.g., PI3K/Akt/Raf1/MEK/ERK [129,130], PI3K/Akt/mTOR [131], and PI3K/Akt/ERK1-2/NF-κB [132]. Furthermore, the flavonoid irigenin targets the TRAIL signaling pathway leading to apoptosis in gastric cancer, with the enhancement of FAS-associated protein with death domain (FADD), death receptor 5 (DR5) and Bax proapoptotic proteins [133], a way targeted also by pinostrobin [134], apigenin [135], kaempferol [136].

**Figure 1:**
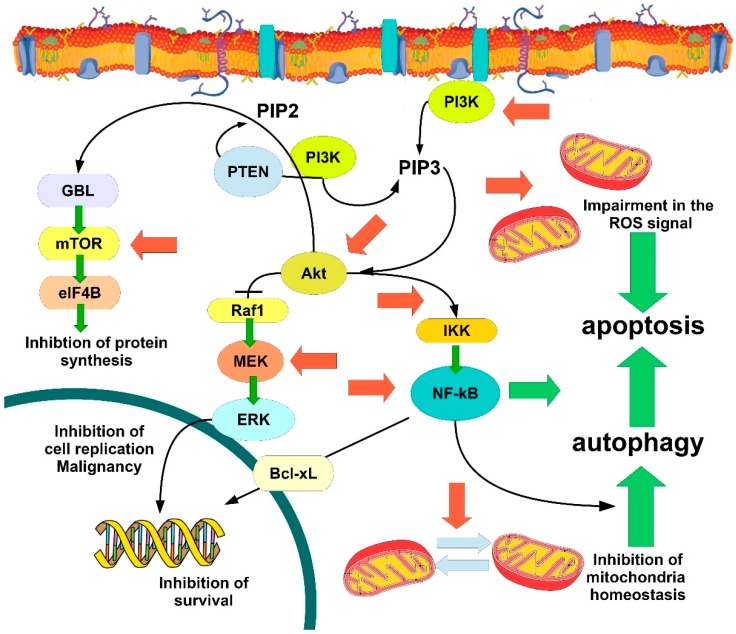
Cartoon showing the major signaling pathways targeted by phytochemicals in cancer. Phytochemicals principally act as inhibitors which red arrows indicate in the PI3K/Akt signaling pathway. Green arrows indicate the action promoted or activated by flavonoids, also following the inhibitory signaling cascades. For details and acronyms, see the text.

Figure 1 shows the major pathways targeted by flavonoids, coumarins and stilbenes (resveratrol) in inducing apoptosis in cell cancer.

### 2.2. Autophagy and the Mitochondria–ER Stress Relatonships Leading to Cancer Cell Death

Flavonoids can target the autophagic machinery in cancer cells, so managing and ruling the survival homeostatic processes yet often leads to the apoptotic destiny of tumoral cells [116]. For example, kaempferol induces autophagy to upregulate miR-340 in human lung cancer cells [41] also via the IRE-JNK-CHOP pathway [137]. The widest activity of flavonoids on apoptosis in cancer cells via the major survival signaling pathways would raise the question about which relationship does exist between autophagy and apoptosis [138], although a fundamental link with the Akt signaling pathway has been recently highlighted [139] and some major components of the fission and fusion machinery in mitochondria, i.e., dynamin-related protein 1 (Drp1), mitofusin1/2 (Mfn1/2) and Optic Atrophy 1 (OPA1), were reported to be involved in the mitochondria-related modulation of autophagy, besides apoptosis and necroptosis [140]. Flavonoids might act in the crosstalk leading to the autophagy–survival mechanism or the autophagy–apoptosis decision [141,142,143], where a possible role is exerted by beclin-1, Bcl-2 and Bcl-xL [144,145]. Furthermore, the regulation of mitophagy and autophagy depends on acetylation (see acetylCoA) and Lys acetylation in mitochondria, accounting for a stringent control in nutrients uptake [146,147]. Autophagy can participate in cancer eradication; for example, silibinin induces MCF7 breast cancer cells death via the upregulation of beclin-1 and Atg12–Atg5 formation, the conversion of the light chain 3 (LC3)-I to LC3-II, mitochondrial leakage of the mitochondrial transmembrane potential and a decline in ATP levels, together with a great increase in ROS [148].

The role of autophagy in cancer has been extensively reviewed [149,150]. Autophagy is a key regulator of cell survival and homeostasis and past reports suggested its role as a tumor suppressor. However, a hypothesis by which autophagy leads to tumorigenesis has been related to its reduction in the stress response capability, thus leading to much more support in cell metabolism and survival processes [149]. Cancer cells are more dependent on autophagy than other cells. The abnormal increase in the metabolic demand shifts the autophagy towards mechanisms, leading to apoptosis or cell cycle arrest. Particularly for RAS-driven cancers, recent reports suggest that they are “autophagy-addicted” [151,152]. Interestingly, the flavonoid 6-C-(E-phenylethenyl)naringenin (6-CEPN), which can be found in naringenin-fortified fried beef, induces cytoprotective autophagy in colon cancer cells, although it inhibits cell growth by dampening the expression of autophagy proteins Atg7 and beclin-1. The compound 6-CEPN strongly activates RAS [153]. Further flavonoids kill cancer cells via an autophagic signaling pathway. The flavone isoorientin induces autophagy in hepatoblastoma cancer, activating ROS signaling leading to p53 expression and so concurrently activates apoptosis by the PI3K/Akt, JNK and p38MAPK signaling pathways [154]. The induction of ROS signaling to result in cancer cell apoptosis, via the initial autophagic process, has been reported also for the flavonoid wogonoside in human glioblastoma cells [155]. Interestingly, it seems that flavonoids do not exert their acknowledged antioxidant potential in cancer; however, a possible reason is that they shift the hormetic curve of survival homeostasis in cancer towards an autophagy-driven apoptotic signal, instead of an autophagy-driven survival process.

Possible sensitive targets of this “pro-oxidant” flavonoids-mediated mechanism are mitochondria, often in association with ER stress and the unfolded protein response (UPR) [156].

The prenylated flavonoid morusin induces an increase in the levels of mitochondrial calcium ions, with ER stress, induction of ROS and loss of mitochondrial membrane potential in epithelial ovarian cancer [157]. Calcium overload in mitochondria induces mitochondrial swelling and dysfunction, leading to morusin-induced paraptosis-like cell death [157]. Paraptosis is a mechanism leading to tumoral cell death, which is different from apoptosis and necrosis [158]. Some flavonoids induce paraptosis in cancer cells. For example, the polyphenol xanthohumol induces paraptosis in leukemia cells via the p38MAPK pathway [159], hesperidin, likewise morusin, causes paraptosis in HepG2 cells by inducing mitochondria calcium overload and subsequently mitochondria dysfunction [160]. Moreover, a possible finely controlled tuning of the cell survival/cell death homeostasis should explain terms such as paraptosis and necroptosis and their relationship with autophagy [161].

Flavonoids might work in the crosstalk between survival and cell death, acting either as anti-oxidant or pro-oxidant molecules, depending on the stress-responsive endowment of the perturbed cell. Certainly, the mitochondrial/ER proteasome orchestrated function may play a major role [17,53,162,163,164,165].

### 2.3. Epithelial Mesenchymal Transition (EMT) and Cell Cycle Arrest

Many phytochemicals, such as flavonoids, induce cancer cell death by targeting the cellular EMT pathways. Nobiletin, a hexamethoxyflavone, inhibits the EMT process initiated by hypoxia in renal cell carcinoma via the NF-κB and the Wnt/β-signaling pathways, dampening cancer migration and invasion (malignancy) [166]. The effect on EMT and therefore on cancer migration can be also associated with the induction of apoptosis [167] and even initially targeting the same signaling systems then leads to apoptosis, such as the Akt-related pathways. For example, apigenin inhibits non-small cell lung cancer metastasis by dampening the EMT signal via a CD26-Akt-Snail/Slug signaling pathway [168]. Furthermore, the PI3K/Akt signaling pathway can lead to cell cycle arrest in cancer [101]. The chalconoid cardamonin induces apoptosis and cell cycle arrest in breast cancer by downregulating Wnt3a/β-catenin induction of EMT, blocking EMT and dampening the metastatic signal [169]. Despite the attempt to induce cell survival, for example, by phosphorylating some signaling molecules, such as ERK, cancer cells can undergo cytotoxicity from flavonoids, which inhibits EMT-driven metastasis and blocks cell cycle [170]. The activity held by phytochemicals is fundamentally made of a pro-toxicant nature; normal cells have the ability to counteract their pro-oxidant potential, whereas cancer cell do not.

ROS are closely associated with EMT in cells and redox regulation in the EMT-related cancer progression is an issue of utmost interest [171,172,173]. One could think that flavonoids may even “use” their antioxidant ability to inhibit the ROS-induced EMT in cancer. However, it has been recently reported that superoxide dismutase promotes EMT, enhancing tumor metastasis, in pancreatic cancer cells via the H_2_O_2_/ERK/NF-κB signaling axis [174]. It is particularly difficult to associate flavonoids with their chemopreventive and anticancer potential by simply retrieving this relationship from their widely reported antioxidant ability. A possible master tuner is the mitochondria oscillatory balance of their biogenesis, which rules any survival control and also affects EMT [175,176,177]. ROS might exert a fundamental role in balancing signals. When cells are metabolically stressed and are driven towards mitochondria impairment, risk of mitochondria calcium overload, ER stress with UPR and so on, mild stress with ROS should work as a signal to induce survival but simply block or interrupt the autophagy, mitophagy or EMT pathway, leading to apoptosis or cell cycle arrest. When cell metabolism and bioenergetics are homeostatically controlled, mild ROS induces the survival process and restore the homeostatic balance. In the case of cancer cells, the homeostatic response is held by an unusual autophagy–survival relationship driven by ROS distress and by EMT. Mild stress (eustress), breaking down this “insane” equilibrium, should lead to cell death and EMT inhibition.

### 2.4. Insights into the Capability of Raw Food and Plant Extracts Containing Phytochemicals to Prevent and Counteract Cancer

Phytochemicals’ ability to counteract cancer has been particularly stressed in current in vitroand in vivoresearch [178]. The regular intake of food edible plants has been long associated with a reduction in the risk to develop cancer. A bulk of more than 200 research investigations can be retrieved from current scientific literature reporting the association between vegetables and fruit intake and the prevention of the most common and diffused cancers in humans, including the recent extensively reviewed research [179]. According to this study, the protective effect of plant-derived substances in daily diet, expressed as relative risk, was assessed for at least 128 of 156 nutritional investigations [179]. For the majority of the investigated cancers, people consuming moderate or poorly enriched diets with fruits or vegetables underwent a doubled risk to develop cancer compared to people with dietary habits including plant-derived raw foods, even considering statistics with confounders. In the case of lung cancer, at least 24 of 25 studies retrieved in the latest years reported protection from plants, after removing confounders for smoking [179]. About 28 of 29 papers reported that fruit consumption reduced the incidence of cancers in oral cavity, esophagus and larynx, 26 of 30 papers reported the fruit consumption decreased the incidence of pancreas and stomach cancer, and 28 of 38 papers revealed the decrease of bladder cancer and colorectal cancer by fruit intake [179]. Furthermore, about 11 of 13 studies’ results are positive for endometrial, cervix and ovary cancers, while the protective effect of fruits and vegetables intake was assessed also for breast cancer in a performed meta-analysis [179]. These data suggest that good cancer preventions should ask for the daily intake of plant-derived raw food [179]. Despite the many positive and encouraging results in in vitro investigations with purified, aglycone flavonoids, raw food is endowed with an additive and synergistic action of the many different phenolic components in the raw matrices, showing a better efficacy in preventing cancer [5].

Flavonoids, as a major class of phenolic compounds, exhibited highly antioxidant activity. These compounds have been connected to reduce the risk of main chronic diseases and have been recognized largely in fruits, vegetables, and other plant foods [180]. For example, indigenous individuals living in Siberian areas possess a typical socio-economical and anthropological tradition, which allow the people to use a particular kind of diet with plant raw foods. Phenolic, flavonoids, elements and organic acids content, antioxidant and cytotoxic activity of six Siberian indigenous fruits have been recently studied [181]. Cell cytotoxicity of the human prostate cancer cell line Du-145 was performed by treating the cell line with at least six different plant extracts of Siberian fruits, encompassing the concentration range of 0–100 μg/mL. Siberian apricot, Siberian mountain ash and Siberian bird cherry showed a moderate cytotoxic effect in a dose-dependent fashion. Siberian apricot extracts caused 50% of the cytotoxic effect at 25 μg/mL and almost a 100% effect at 100 μg/mL [181].

Some enzymes are particularly targeted by flavonoids contained in Siberian fruits. For example, cyclooxygenases (COXs), which play a major role in inflammatory reactions and carcinogenesis, besides modulating cell apoptosis, proliferation and the angiogenic mechanisms in tissues, are possible targets of these flavonoids. For example, anthocyanins in cherry fruits target COXs, exhibiting an anti-inflammatory activity [182]. Cherry extracts are considered as a good candidate to inhibit COX proinflammatory and pro-carcinogenetic activities. With their sweet and sour taste, cherry fruits are an acknowledged food used by Siberian to promote their good health. Cherries contain a huge amount of anthocyanins and anthcyanidins, mixed with phenolic acids. The frequent and regular intake of these fruits appears particularly beneficial in reducing the incidence of certain cancers. The study on lipid peroxidation and on the activity of COXs by flavonoids from different sweet and sour varieties of Siberian cherries assessed the role of these compunds as natural inhibitors [182]. The role of cherry anthocyanins for colon cancer prevention was confirmed in another study using a combination of dietary anthocyanin-rich extract and suboptimal dosages of sulindac in mice for 19 weeks [183]. Cherry leaves alcohol extract containing flavonoids showed a pronounced anti-inflammatory activity (52.1%), not inferior to the compared drug quercetin (50.9%) [184].

The cancer suppression activity of peach and plum extracts were also evaluated [185]. It was found that within all fractions, flavonoids and procyanidins were more potent against the three cell lines investigated in the study. Quercetin 3β-glucoside was the most bioactive compound identified in the chromatographic fractions, able to exert an anti-proliferative activity against MDA-MB-435 and MCF-10A breast cancer cell lines [185]. Growth-inhibitory effects were also reported on Caco-2, SW1116, HT29 and NCM460 cancer cell lines from extracts of plums and peaches notoriously being enriched with anthocyanins [186]. The most efficacious fraction contained flavonoids rather than anthocyanins, suggesting that a synergistic activity with phenolics in plum and peaches extracts from Siberian fruits should explicate the action on cancer cell lines previously observed [186].

Many recent research investigations have shown that phytochemicals in fruits express an orchestrated panoply of similar actions, often shown as overlapping effects, which include the regulation of the expression of detoxyfying antioxidant enzyme complexes, activation of innate immunity, modulation of gene expression, cell replication, autophagy and apoptosis and antimicrobic activity [5]. Some examples are particularly interesting for the effect of oxidation. *Prunus domestica* leaves extract is rich in hydroxycinnamic acids and flavonoids. The extract was tested in vitro and showed the greatest activity against the suppression of lipid peroxidation. It was found that the extract reduced the level of peroxidation products by 88.1% [187].

A prospective study, enrolling about 9959 subjects (age: 15–99 years, sex: equally distributed) and performed in Finland, reported an interesting inverse association between flavonoid assumption with diets and incidence of cancer [5].

Following a 24-year follow-up period, researchers reported that the risk of lung cancer was reduced by 50% (superior quartile of the flavonoid intake amounts). The consumption of quecetin contained in apples and onions in Hawaii was found to be inversely corelated with the incidence of lung cancer.

Moreover, the effect of onions intake resulted particularly promising in reducing squamous cell carcinoma incidence [5]. The increasing plasma levels of quercetin from ingested onions were also associated with an enhanced resistance to DNA strand breakage occurring in lymphocytes and a reduction in the level of some catabolites from oxidative stress in urine samples [5].

Further reports showed also that anthocyanins and cyanidins are able to reduce colon cancer cell lines HT29 and HCT 116 [188]. These studies reported that the 50% inhibitory concentration (IC_50_) values of anthocyanins and cyanidins were780 mM and 63 mM for HT29 cells and 285 mM and 85 mM for HCT116 cells, respectively, suggesting that anthocyanins and cyanidins in cherries may be promising as anticolon cancer natural compounds [188]. Actually, anthocyanidins counteract a lot of biological mechanisms, leading to cancer aggressiveness, including metastasis and chemoresistance [189].

Cyanidin is a polyphenolic pigment that can be easily found in many red berries, including blackberry, grapes, raspberry, cranberry, besides plums, apples and red cabbage and it is present also in red onions. It has a good potential benefit against cancers, thanks to its antioxidant property [178]. Cyanidin has been reported to dampen cell proliferation in cancer and to inhibit *COX-2* and *iNOS* gene expression in colon tumors [190,191,192,193]. A study showed that cyanidin-3-glucoside inhibited the benzo[a]pyrene-7,8-diol-9,10-diol-epoxide-induced activation of the transcription factors AP-1 and NF-κB, including the phosphorylation of MEK, MKK4, Akt and MAPKs [194]. Furthermore, they also blocked the activation of the Fyn kinase signaling pathway, leading to a chemopreventive potential [194]. Further flavonoids, such as cyanidin-3-O-glucoside and cyanidin-3-O-rutinoside, as well as the ethanol extracts of raspberries, caused the inhibition of the cellular growth in highly tumorigenic rat esophagus cell lines RE-149 DHD but not in the weaker tumorigenic cell line RE-149 [195]. Further results showed that cyanidin inhibits ultraviolet light B UVB-induced *COX-2* and *PGE2* expression in epidermal cells by suppressing the MAPK-mediated NF-κB and AP-1 activity [195]. Furthermore, cyanidin is able to target MKK-4 MEK-1 and Raf-1 in the suppression pathway of UV-induced COX-2 [195]. Additionally, cyanidin-3-galactoside and cyanidin-3-glucoside are substrates for BRCP, while cyanidin, cyanidin-3,5-diglucuronide and cyanidin-3-rutinoside are BRCP inhibitors, although their effect on MDR1 is weak [178,196].

Catechins in green tea represent another hallmark of anticancer flavonoids. Green tea is a very widespread and commonly used beverage in the world. The majority of anticancer effects from green tea are attributed to EGCG [197,198]. EGCG is able to kill cancer cells notoriously resistant to proapoptotic stimuli and induces death through a necroptosis pathway in many cancer [189].

Tea polyphenols can prevent cancer by modulating epigenetic aberrations taking place in DNA methylation, histone modifications, and microRNAs. By altering these epimutations, they regulate chromatin dynamics and expression of those genesinducing or suppressing cancer formation [190].

Both theaflavins and thearubigins, which are very abundant polyphenolic compounds in black tea, possess a strong anticancer potential. Polyphenols in black tea inhibit cell proliferation and trigger apoptosis in Du 145 prostate cancer cells [191].

Theuse of flavonoids from raw fruits and vegetables should be encouraged with respect to nutraceutcals from purified active substances [192]. Effects of plant flavonoids on cancer can be even protective towards cancer cell lines; effects of various polyphenols, such as catechins, quercetin, flavanones, isoflavones, ellagic acid, lignans, polyphenols from red wine such as resveratrol, and curcumin, on various type of cancers, including mouth, stomach, duodenum, colon, liver, lung, mammary gland and skin, were observed [191]. This should account for the bimodal activity held by flavonoids.

The flavonol quercetin usually acts as an anticancer molecule via a process that involves the downregulation of some oncogenes (e.g., Mcl-1, Ras, MEK, P3K) or also the upregulation of some tumor suppressor genes, leading to the eradication of cancer [193]. Kaempferol, which can be found in vegetables such as broccoli, tea, grapefruit, Brussels sprouts, witchhazel and apples, has been reported as efficacious in pancreatic and lung cancer [199,200,201]. It has antiangiogenic and scavenging effects of oxygen radicals [178,202,203,204].

The flavone fisetin, which is present in plants, such as strawberries, persimmon, apples, *Acacia greggii*, *Acacia berlandieri*, Euroasiansmoketree, parrot tree, onion, cucumber and grape, is a strong antioxidant, modulating protein kinase and lipid kinase pathways [205]. Fisetin has been found to exert antitumoral actions in HCT-116 colon cancer cell lines [178]. Together with other flavones and flavonoids such as luteolin, galangin, quercetin and EGCG, fisetin induces the expression of the Nrf2 factor; moreover, it induces the phase II gene product *HO-1* in human retinal pigment epithelial (RPE) cells. These cells may protect RPE cells from oxidativestress-induced death, with high efficacy and negligible toxicity [206]. Moreover, itlowered hydrogen peroxide (H_2_O_2_)-induced cell death [189,207].

The isoflavone genistein is present in plants, such as lupine, fava beans, soybeans, kudzu, and psoralea, *Flemingiavestita*, and coffee. It is an antioxidant and an anthelmintic, besides exerting antioxidant effects and antiangiogenic actions (blocking formation of new blood vessels), and also blocking cell replication and survival [178].

The flavanone naringenin, commonly found in citrus fruit and oranges, besides tomatoes skin and grapefruit, has a potent antioxidant property [208,209]. It suppresses the TGF-β ligand–receptor interactions [208], and TGF-β signaling, controlling a various group of cellular mechanisms in cancer, including cell proliferation, differentiation and apoptosis, as well as morin [189,208,210].

## 3. Conclusions and Future Remarks

The use of flavonoids and other phytochemicals in cancer research still remains a promising expectation, despite the many controversial aspects regarding the yet currently paucity in clinical results. Nutritional panels and dietary guidelines should encourage and support the introduction of plant phenolics in the daily meal composition, try to possibly reduce the amount of synthetic nutraceuticals and instead promote raw vegetables and fruit intake. Supplementation may be desirable if the composition formula is the closest possible with the raw source of the supplemented phytochemicals, a compulsory item that is very difficult to reach with optimal results. Anyway, research on flavonoids and plant extracts ensures current pharmacology to find and retrieve new drugs, able to specifically target cancer cells and to update our therapeutic endowment against malignancies. The very recent novelties in the field of polyphenol research against cancer refer to not only microRNA but also long non-coding RNA, such as glyceolinoccurring in soybean [211]. New fundamental insights in molecular biology of phytochemicals can improve our ability to investigate and use these compounds against cancer.

One fundamental question could be: why is using flavonoidsmore beneficial than using chemopreventive drugs against cancer? As a future perspective, flavonoids act as natural molecules, with surprisingly fewer side effects than chemopreventive drugs. The ability to sensitively tune fundamental mechanisms in cell survival, autophagy and apoptosis renders these compounds particularly useful in being adopted as prodrugs and also therapeutic molecules against cancer. Clinical research must be improved in this sense, in order to retrieve a more and more outstanding data collection about the excellent ability of these various natural substances to prevent and fight tumors in humans.

## Figures and Tables

**Table 1:** Some of the very recent examples of the roles exerted by flavonoids in cancer cells.

Classification	Compound	Activity		References
Flavones 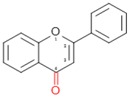	Apigenin	↑	Anticancer activity via the Wnt/β-catenin pathway and JAK-STAT. Induction of apoptsis in TRAIL-resistant cancers	[60,61,62,63,64]
	Luteolin	↑	Induction of apoptosis and autophagy in ANA-1 cells via the p38, JNK and Akt signaling pathways, inhibiting Bcl-2 and beclin-1 and activating caspase-3 and caspase-8	
		↓	Proliferation of BT474 and MCF-7 breast cancer cells	
		↑	Apoptosis in BT474 and MCF-7 breast cancer cells	
		↑	Apoptosis in ACS gastric cancer	
	Tangeritin	↓	Cell cycle in MCF7 and MDA-MB-468 breast cancer cells via the CYP1A1/CYP1B1-mediated metabolism	
Flavonols 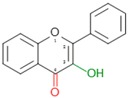	Quercetin	↓	Many types of cancer via apoptosis and inhibition of cell replication	[41,65,66,67,68,69,70,71]
	Kaempferol	↑	Apoptosis and autophagy in human lung cancer cells A549 via upregulation of miR-340	
		↑	Apoptosis in HCT116, HCT15, and SW480 colorectal cancer cells	
	Myricetin	↓	Prostate cancer cell metastasis by cytotoxic activity	
	Fisetin	↓	Growth and metastasis and EMT in MDA-MB-231 and BT549 breast cancer cells	
	Galangin	↓	Proliferation of human kidney A498 cancer cells by the induction of apoptosis-targeted PI3K/Akt/mTOR signaling	
	Isorhamnetin	↓	Growth of MCF-7 breast cancer cells	
Flavanones (citrus fruit flavonoids) 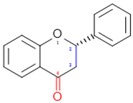	Hesperetin	↑	Apoptosis in H522 lung cancer cells	[72,73]
	Naringenin	↓	Prostate cancer metastasis via voltage-gated sodium channel blockage	
Flavanonols 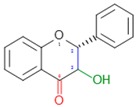	Taxifolin	↓	Mammary carcinogenesis via the LXR-mTOR/Maf1/PTEN axis and the CYP1A1- and CYP1B1-mediated cancer	[74,75]
Flavans (Flavanols) Green tea catechins 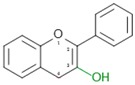	Epigallocatechingallate (EGCG)	↑	Chemoprevention in glioblastoma	[76,77,78,79,80]
		↑	Apoptosis in chronic myeloid leukemia by Bcr/Abl-mediated p38-MAPK/JNK and JAK2/STAT3/AKT signaling	
	Catechin, EGCG	↓	Lung tumor growth via the inhibition of programmed cell death-ligand1 (PD-L1)	
	Epicatechin-3-*O*-gallate (ECG)	↓	LNCaP and PC-3 prostate cancer cell growth	
	Epigallocatechin (EGC)	↓	Suppression of HPV and tumors with curcumin and resveratrol	
Anthocyanidins 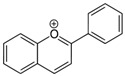	Cyanidin	↓	Angiogenesis in breast cancer via the STAT3/VEGF pathway and miR124 mediated STAT3 downregulation	[81,82,83]
	Delphinidin	↑	Apoptosis and autophagy in HER-2 positive breast cancer MDA-MB-453 and BT474 cells	
		↑	Apoptosis and EMT in human osteosarcoma cell lines via the ERK2/p38MAPK pathway	
Isoflavonoids 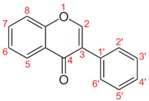	Genistein	↑	Apoptosis in Mcl1 human laryngeal cancer cells	[84,85,86,87]
		↓	Proliferation of EP3-expressing melanoma	
			Alters epigenetic in MDA-MB-231 breast cancer cells	
	Daidzein	↑	Apoptosis in colon cancer cells	

Dow arrows = inhibition; Top arrows: activation and/or promotion. Red colours in chemical formulas indicate the typical functional groups and/or chemical positions for typical attachments of substituents (isoflavonoids).

## Abbrevations

AAK	Akt-activated kinase
Akt	protein kinase B
AMPK	adenosine-monophosphate-activated proteinkinase
ARE	antioxidant-responsive element
ASK1	apoptosis signal-regulating kinase 1
Atg12–Atg5	autophagy factor12–autophagy factor5
Atg7	autophagy factor 7
Bax	Bcl-2-associated X protein
Bcl-2	B-cell lymphoma 2 (an antiapoptotic protein)
Bid	BH3-interacting domain
BCRP	breast cancer resistance protein
BNP3	Bcl-2-adenovirusE1B 19 kDaprotein-interactingprotein3
6-CEPN	6-C-(E-phenylethenyl)naringenin
Cip1	cyclin-inhibitor protein 1 (p21)
CYP	cytochrome P450
DAF16	Dauer-associated factor 16
DAPK2	death-associated protein kinase 2
DRP1	dynamin-related protein 1
ERK	extracellularsignal-regulated kinase
FOXO	forkhead box O3
HO-1	heme oxygenase 1
IKK	IκB kinase
iNOS	inducible nitric oxide synthase
IRE-JNK-CHOP	iron response element- Janus kinase-cyclophosphamide-hydroxydaunorubicin-oncovyn-prednisone)
JAK2	Janus kinase 2
JNK	Janus kinase
p38MAPK	p38 mitogen-activated protein kinase
MEK	a synonymof MAPK
MDR1	multidrug resistant 1
Mfn2	mitofusin-2
mTOR	mammalian target of rapamycin
NB4	an acute promyelocytic leukemia cell line
NF-κB	nuclear factor kappa-light-chain-enhancer of activated B cells
Nrf2	nuclear factor erythroid 2-related factor 2
PARP	poly-ADP-ribose polymerase
PERK–ATF4	protein kinase RNA-like endoplasmic reticulum kinase–activating transcription factor 4
PGC1α	peroxisome proliferator-activated receptor gamma coactivator 1-alpha
PGE2	prostaglandin E2
pEGFR	phosphorylated epithelial growth factor receptor
PKA-PI3K	protein kinase A-phosphoinositide 3 kinase
Raf1	Ras-associated factor 1
SIR 2-1	sirtuin 2-1
SIRT1	sirtuin 1
Snail/Slug	transcription actors in EMT
STAT3	signal transducer and activator of transcription 3
TFAM	transcription factor A, mitochondrial
TRAIL	TNF-related apoptosis-inducing ligand
Wnt	wingless/integrated
